# Cycloheximide Can Induce Bax/Bak Dependent Myeloid Cell Death Independently of Multiple BH3-Only Proteins

**DOI:** 10.1371/journal.pone.0164003

**Published:** 2016-11-02

**Authors:** Katharine J. Goodall, Megan L. Finch-Edmondson, Joanne van Vuuren, George C. Yeoh, Ian E. Gentle, James E. Vince, Paul G. Ekert, David L. Vaux, Bernard A. Callus

**Affiliations:** 1 Department of Biochemistry, La Trobe Institute for Molecular Science, La Trobe University, Bundoora, VIC, Australia; 2 School of Chemistry and Biochemistry, University of Western Australia, Crawley, WA, Australia; 3 Harry Perkins Institute of Medical Research, QEII Medical Centre, Nedlands and Centre for Medical Research, the University of Western Australia, Crawley, WA, Australia; 4 Institute for Medical Microbiology and Hygiene, Hermann-Herder-Str. 11 79104 Freiburg Germany; 5 The Walter and Eliza Hall Institute of Medical Research, Parkville, VIC, Australia; 6 Department of Medical Biology, University of Melbourne, Parkville, Victoria, Australia; 7 Murdoch Childrens Research Institute, Royal Children's Hospital, Parkville, VIC, Australia; 8 School of Health Sciences, The University of Notre Dame Australia, Fremantle, WA, Australia; Roswell Park Cancer Institute, UNITED STATES

## Abstract

Apoptosis mediated by Bax or Bak is usually thought to be triggered by BH3-only members of the Bcl-2 protein family. BH3-only proteins can directly bind to and activate Bax or Bak, or indirectly activate them by binding to anti-apoptotic Bcl-2 family members, thereby relieving their inhibition of Bax and Bak. Here we describe a third way of activation of Bax/Bak dependent apoptosis that does not require triggering by multiple BH3-only proteins. In factor dependent myeloid (FDM) cell lines, cycloheximide induced apoptosis by a Bax/Bak dependent mechanism, because *Bax*^*-/-*^*Bak*^*-/-*^ lines were profoundly resistant, whereas FDM lines lacking one or more genes for BH3-only proteins remained highly sensitive. Addition of cycloheximide led to the rapid loss of Mcl-1 but did not affect the expression of other Bcl-2 family proteins. In support of these findings, similar results were observed by treating FDM cells with the CDK inhibitor, roscovitine. Roscovitine reduced Mcl-1 abundance and caused Bax/Bak dependent cell death, yet FDM lines lacking one or more genes for BH3-only proteins remained highly sensitive. Therefore Bax/Bak dependent apoptosis can be regulated by the abundance of anti-apoptotic Bcl-2 family members such as Mcl-1, independently of several known BH3-only proteins.

## Introduction

The role of Bcl-2 as an inhibitor of cell death was first established in FDC-P1 cells, an IL-3 dependent mouse myeloid cell line [1]. These cells undergo apoptosis when growth factor is removed, but when growth factor was removed from cells over-expressing Bcl-2, they arrested, but did not die.

Similar factor dependent myeloid (FDM) cell lines have been generated by infecting murine bone marrow or foetal liver cells with retroviruses expressing HoxB8, and culturing in IL-3 [2–5]. FDM lines lacking genes for pro-apoptotic members of the Bcl-2 family, such as the multi-domain proteins (Bax and Bak), or a number of BH3-only proteins, have been generated in a similar way by using bone marrow or foetal liver from gene deleted mice. In this way we have obtained IL-3 dependent myeloid lines lacking genes for Bax or Bak, Bax and Bak, Blk (Bik), Puma, Noxa, Bim, Bad, Bim, Bmf and Hrk as well as lines lacking both Bim and Bad, both Bim and Bid and both Puma and Noxa. All of these cell lines remain dependent on cytokines for growth and proliferation.

Cycloheximide (CHX) is an inhibitor of protein synthesis [6]. Many cell types rapidly undergo apoptosis when exposed to CHX. In FDC-P1 cells, CHX-induced apoptosis is mediated by Bax and/or Bak because it can be inhibited by over-expression of Bcl-2 [7].

Bax/Bak dependent apoptosis is widely believed to be triggered by BH3-only proteins and that they have an essential and obligatory role in the activation of Bax and/or Bak [8, 9]. The BH3-only members such as Bik, Bid, Bim, Bad, Puma, Noxa, Bmf and Hrk fall into two classes. The “direct activators”, such as Bid, Bim and Puma, can bind directly to Bak or Bax to activate them. Members of the other class, the “indirect activators”, which includes Bad, Bik, Bmf, Hrk and Noxa, act by binding to anti-apoptotic Bcl-2 family members (namely Mcl-1, Bcl-2, Bcl-x, A1 and Bcl-w) and thereby prevent them from inhibiting Bax or Bax [10].

To determine which BH3-only protein(s) were responsible for apoptosis of FDM cells in response to cycloheximide, we compared the sensitivity of cell lines mutant for various pro-apoptotic Bcl-2 family members.

We were surprised to find that none of the lines lacking genes for individual BH3-only proteins were resistant to CHX induced apoptosis, and furthermore, lines lacking both Bim and Bid, and with undetectable levels of Puma [4], still underwent apoptosis in response to CHX.

Collectively these results indicate that CHX does not induce FDM cell death by activation of BH3-only proteins, but that activation of Bax/Bak and apoptosis in this case is caused by a reduction in the abundance of Mcl-1. Furthermore, they suggest that loss of one or more pro-survival proteins can be sufficient to permit activation of Bax/Bak, and that in some circumstances Bax and Bak can be activated in the absence of BH3-only proteins involvement.

## Results

**Fig 1:**
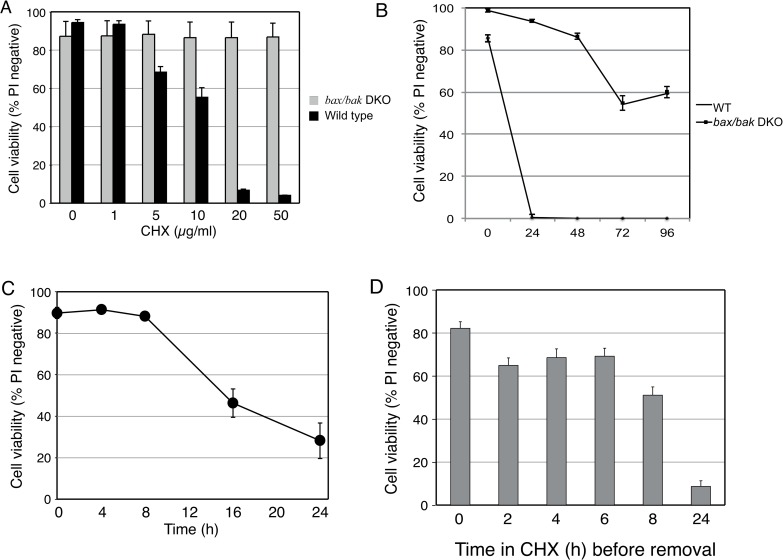
CHX induces a time and dose-dependent Bax/Bak-dependent death in FDM cells. A) WT and *bax/bak* DKO cells were treated with CHX at the concentrations indicated for 24 h before determining cell viability by PI exclusion and flow cytometry. *bax/bak* (B) and WT (C) cells were treated with 20 μg/ml CHX for the times indicated before cell viability was determined. D) WT cells were incubated with 20 μg/ml CHX for the times indicated before being washed and resuspended in fresh media without CHX. Cultures were continued before cell viability was determined at 24 h. Data presented are expressed as mean ± SEM from 3 independent experiments.

Initially, a dose-response experiment was performed to determine the concentration of cycloheximide (CHX) that caused FDM cells to die. CHX induced a dose-dependent decrease in viability of wild-type (WT) FDM cells for concentrations above 1 μg/ml with greater than 90% of cells killed at 20 μg/ml by 24 h (Fig 1A). CHX induced cell death by this time point was dependent on the expression of Bax or Bak because *bax/bak* deficient FDM cells derived from *bax/bak* double knockout (DKO) mice were profoundly resistant to CHX treatment (Fig 1A). This resistance was confirmed by treating *bax/bak* DKO cells for 96 hours with CHX, at which the majority of the cells were still viable, in contrast to the rapid cell death of the WT cells (Fig 1B) From these experiments a concentration of 20 μg/ml CHX was chosen and was used in all subsequent experiments.

Next, WT cells were treated with CHX for varying times to determine how rapidly it induced cell death. In this experiment, most of the cells had died by 16 h (Fig 1C). To determine whether the effects of CHX treatment were reversible, WT FDM cells were treated for between 2 and 24 h with 20 μg/ml CHX, and then washed and resuspended in CHX-free media, and their viability was measured at 24 h. As seen in Fig 1D, incubating cells for less than 2–6 h with CHX had only modest effects on cell viability measured at 24 h. However, incubating for more than 8 h caused over half the cells to die by 24 h. Collectively, these experiments show that cells treated with CHX do not die immediately, but when exposed for greater than 8–24 h become irreversibly committed to die by a Bax/Bak dependent process.

**Fig 2:**
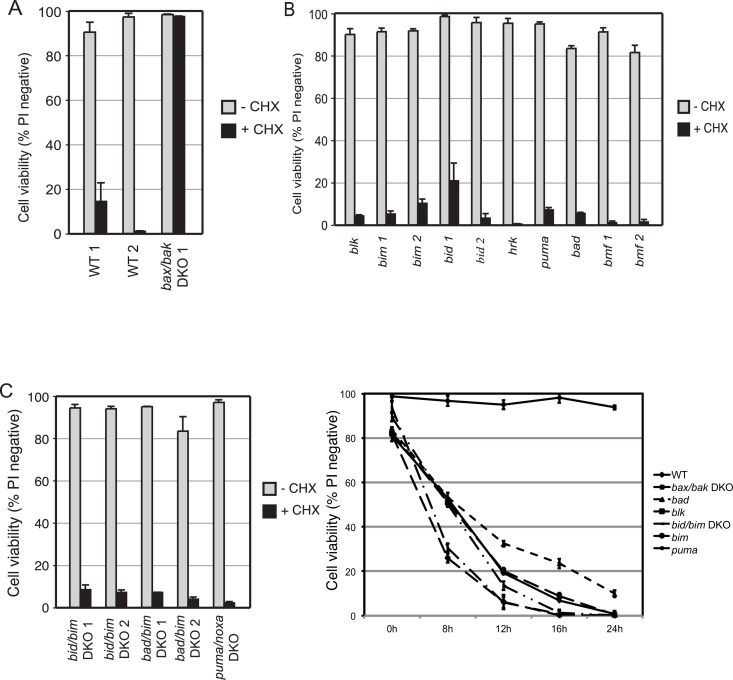
CHX induces death in FDM cells independently of several known BH3-only proteins. Viability of (A) WT and *bax/bak* DKO, (B) single BH3-only knockout and (C) BH3-only DKO FDM cell lines were determined after 24 h of treatment with 20 μg/ml CHX. (D) WT, *bax/bak* and BH3-only knockout cells were treated with 20 μg/ml CHX for the times indicated before cell viability was determined. Data shown is expressed as mean ± SEM from 3 independent experiments. The numbers after a cell line’s name refer to independent clonal lines of the same genotype.

In order to determine the requirement for BH3-only proteins in the activation of Bax/Bak in this system, we examined the ability of CHX to induce death of FDM cell lines derived from mice in which the genes encoding various BH3-only proteins were deleted. Consistent with the previous results, two independent WT clones of FDM cells were highly sensitive to CHX, with a significant loss in viability observed at 24 h post-treatment (Fig 2A). Again *bax/bak* DKO cells were highly resistant to CHX treatment. Next, FDM cells bearing gene deletions for a single BH3-only protein were examined for their sensitivity to CHX treatment. Remarkably, none of the BH3-only single knockout (SKO) cell lines examined exhibited any resistance towards CHX (Fig 2B). One of the *bid* knockout cell lines, *bid 1*, showed slightly increased resistance to CHX-induced toxicity yet the other *bid*^*-/-*^ clone, *bid 2*, did not exhibit the same insensitivity. This suggests that the slight resistance in the *bid 1* line is due to clonal variation.

Next, clones of FDM DKO cells containing combinations of BH3-only protein gene deletions were examined for their sensitivity to CHX. Similar to the SKO results, none of the DKO cells lines examined exhibited any protection towards CHX-induced cell death (Fig 2C). It is also worth noting that despite the *bid 1* cells exhibiting a slight resistance to CHX (Fig 2B), neither of the *bid/bim* DKO cell lines showed a similar resistance (Fig 2C), further suggesting that the slight resistance observed with *bid 1* cells is due to clonal variation. To rule out the possibility that cell lines lacking BH3-only proteins might show resistance to CHX at earlier time points, we performed a time-course experiment using several knockout cell lines. Consistent with previous data, *bax/bak* DKO lines showed profound resistance to CHX (Fig 2D). In contrast, the *bad*, *puma*, *bim* and *blk* SKO lines as well as the *bid/bim* DKO cells lost viability at similar rates to that of WT cells following CHX addition. Although our entire panel of cell lines was not tested, these results suggest that the absence of one of more BH3-only proteins does not prevent or delay activation of Bax/Bak dependent cell death even at early times following CHX treatment.

The uptake of PI into dead or dying cells due to membrane damage is a relatively late event in the apoptotic process. Since the effects of CHX that resulted in cell death occurred between 8–16 h of treatment (Fig 1B), we looked for changes that might lead to activation of Bax or Bak within 8 h of CHX addition. Because the results in Fig 2 argued that none of the members of the BH3-only protein family examined were essential for activation of Bax or Bak, we looked to see whether addition of CHX might trigger apoptosis by causing a reduction in the levels of an anti-apoptotic Bcl-2 family member.

**Fig 3:**
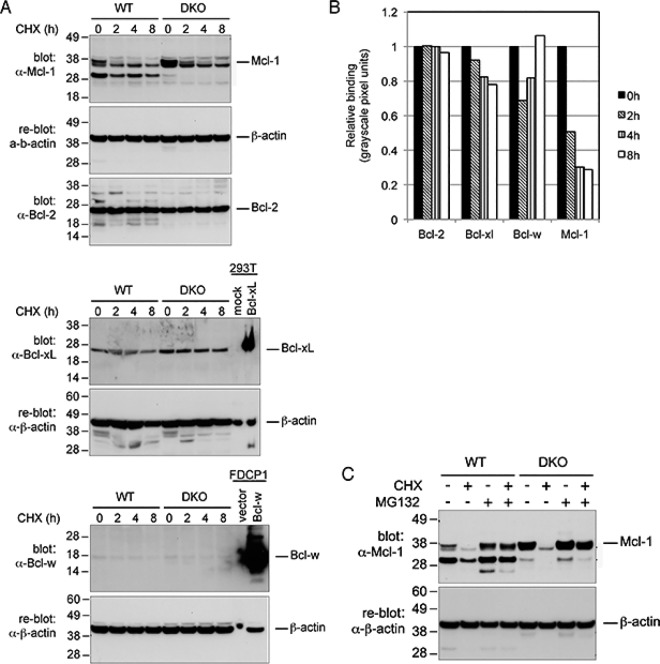
CHX induces the proteasomal loss of Mcl-1, but not other pro-survival Bcl-2 family proteins. A) Lysates of WT and *bax/bak* DKO cells treated with 20 μg/ml CHX for 0–8 h were separated by SDS-PAGE, transferred to membranes and immunoblotted with antibodies to Mcl-1, Bcl-2, Bcl-xL and Bcl-w. 293T cells transiently expressing mouse Bcl-xL, and FDCP1 cells expressing Bcl-w, were used as positive controls for the Bcl-xL and Bcl-w antibodies. ß-actin was used a loading control. B) Densitometry analysis of western blots probed with pro-survival proteins, normalized to 0h timepoint. Mcl-1 densitometry refers only to the transient form of the protein, in *bax/bak* DKO cells. Results are representative of three independent experiments. C) WT and *bax/bak* DKO cells were pre-incubated with or without 20 μM MG132 before being treated with or without 20 μg/ml CHX as indicated. Cell lysates were immunoblotted for Mcl-1 and ß-actin as shown. Asterisks indicate cleavage products of Mcl-1 and cross-reactive species.

We determined the effect of CHX on the levels of the Bcl-2 pro-survival family members in both WT and *bax/bak* DKO cells (Fig 3). The advantage afforded by the *bax/bak* DKO cells is that changes in protein expression can be determined without confounding effects of cell death, such as caspase activation, which occurred in the WT cells. Levels of Bcl-2, Bcl-xL and Bcl-w proteins were not affected by CHX in either WT or *bax/bak* DKO cells within the 8 h time course, indicating these are stable proteins with long half-lives (Fig 3A). Quantification data is of levels of pro-survival proteins in Bax/Bak DKO cells after addition of CHX, only showing the larger, transient band of Mcl-1. A1 was not detected in these cells. Unlike the other pro-survival proteins, the abundance of Mcl-1 rapidly decreased in both WT and *bax/bak* DKO FDM lines. Mcl-1 exists as a doublet, with the smaller band being a more stable form in which the N-terminal tail has been truncated [11, 12]. However, the larger band has a shorter half-life, and in WT cells, this form of Mcl-1 fell to barely detectable levels within 2 h of CHX treatment. In *bax/bak* DKO cells, the abundance of Mcl-1 in untreated cells was much higher than in the WT cells, but the addition of CHX caused a rapid decrease in levels of the unstable form of Mcl-1 such that it was barely detectable within 4–8 h of treatment. These results suggest that loss of Mcl-1 may be responsible for Bax/Bak activation following CHX addition.

To examine whether the loss of Mcl-1 in the presence of CHX was mediated by the proteasome, WT and *bax/bak* DKO cells were treated with CHX for 4 h with or without a 1 h pre-incubation with the proteasome inhibitor, MG132. Treatment of both cell lines with MG132 caused an increase in the levels of Mcl-1, indicating that its abundance in resting cells is limited by turnover within the proteasome (Fig 3C). Consistent with the previous results, addition of CHX alone led to the complete loss of the unstable form of Mcl-1 within 4 hours. Significantly, pre-treatment of cells with MG132 prevented the loss of Mcl-1, indicating that normal proteasomal turnover of Mcl-1 accounts for the loss of Mcl-1 following inhibition of protein synthesis with CHX. As MG132 prevented degradation of Mcl-1 following the addition of CHX, we then examined whether MG132 could prevent CHX-induced cell death in wild-type FDM cells. Unfortunately, treatment with low concentrations of MG132 were highly toxic to the FDM cells, thus this possibility could not be examined in these cells.

**Fig 4:**
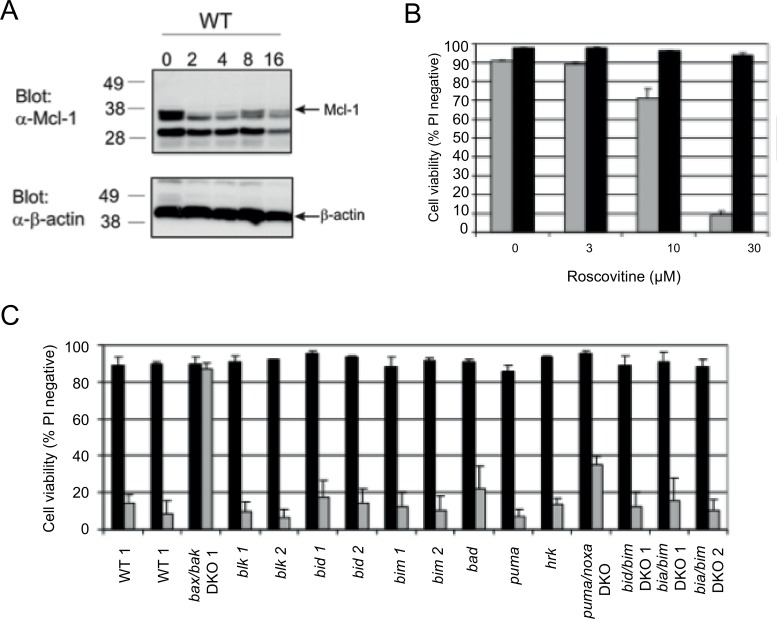
Roscovitine reduces levels of Mcl-1 and induces death in FDM cells independently of BH3-only proteins. A) Lysates of WT and *baax/bak* DKO FDM cells treated with roscovitine (30 μM) for the indicated times were separated by SDS-PAGE, transferred to membrane and immunoblotted with the Mcl-1. β-actin was used a loading control. B) WT cells were treated with increasing concentrations of roscovitine before cell viability was determined. C) WT, *bax/bak* and BH3-only knockout cells were treated with 30 μg/ml roscovitine for 24 h before cell viability was determined. Data shown is expressed as mean ± SEM from 3 independent experiments. The numbers after a cell line’s name refer to independent clonal lines of the same genotype.

The loss of Mcl-1 following CHX treatment suggests that another means of reducing Mcl-1 might also initiate cell death in a BH3-independent manner, similar to CHX. Roscovitine, a CDK inhibitor, has been shown to inhibit Mcl-1 transcription [13]. Consistent with this, the treatment of FDM cells with roscovitine results in the rapid loss of Mcl-1 (Fig 4A). To investigate its effects on viability, cells were then treated with increasing concentrations of roscovitine, which induced profound cell death in a Bax/Bak dependent manner since *bax/bak* DKO cells were highly resistant to its effects (Fig 4B). Next we tested our panel of FDM knockout cell lines with roscovitine, and consistent with out CHX data, none of the BH3-only knockout cell lines showed any significant protection against roscovitine (Fig 4C). These results suggest that, like CHX-induced cell death, the BH3-only proteins are not involved in the initiation of roscovitine-induced FDM cell death but rather it is the loss of Mcl-1 that triggers activation of cell death.

**Fig 5:**
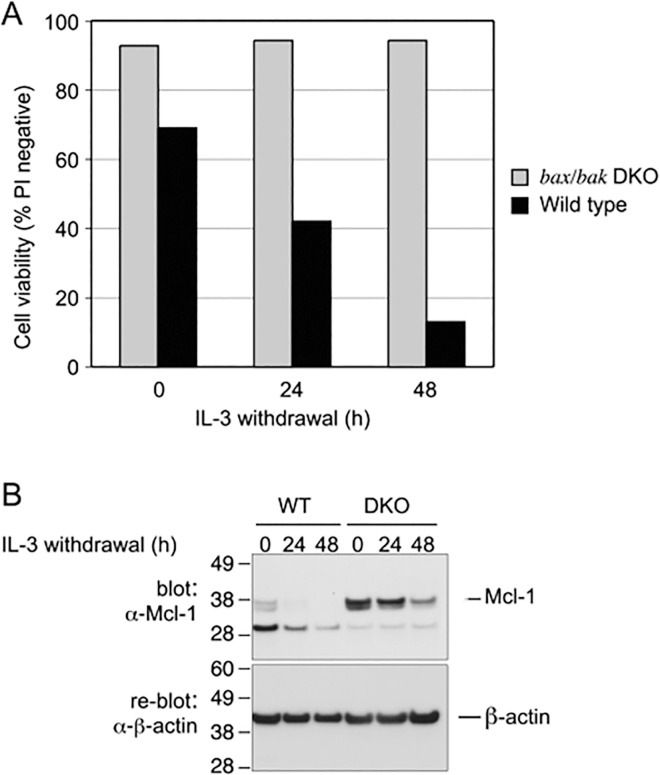
IL-3 withdrawal reduces Mcl-1 abundance. A) WT and *bax/bak* DKO cells were washed and incubated in media without IL-3. Cell viability was determined at 24 and 48 h after IL-3 removal. Data shown are from a single experiment. B) Cell lysates were prepared at 0, 24 and 48 h following IL-3 withdrawal and immunoblotted for Mcl-1 and ß-actin as shown.

As with addition of CHX, withdrawal of IL-3 from the culture media of WT FDM cells causes a loss of viability over 48 h, whereas *bax/bak* DKO cells are completely resistant to this apoptotic stimulus (Fig 5A) [2]. In order to determine whether withdrawal of IL-3 also caused a reduction in Mcl-1, we examined its abundance in lysates of cells at 24 and 48 h after IL-3 withdrawal. Mcl-1 was not detected in WT cells 24 h after IL-3 removal (Fig 5B), but its absence might have been a consequence of cell death, or a cause, or both. In *bax/bak* DKO cells, Mcl-1 levels were reduced, but by a lesser extent, and it was still detectable even at 48 h following IL-3 withdrawal (Fig 5B).

**Fig 6:**
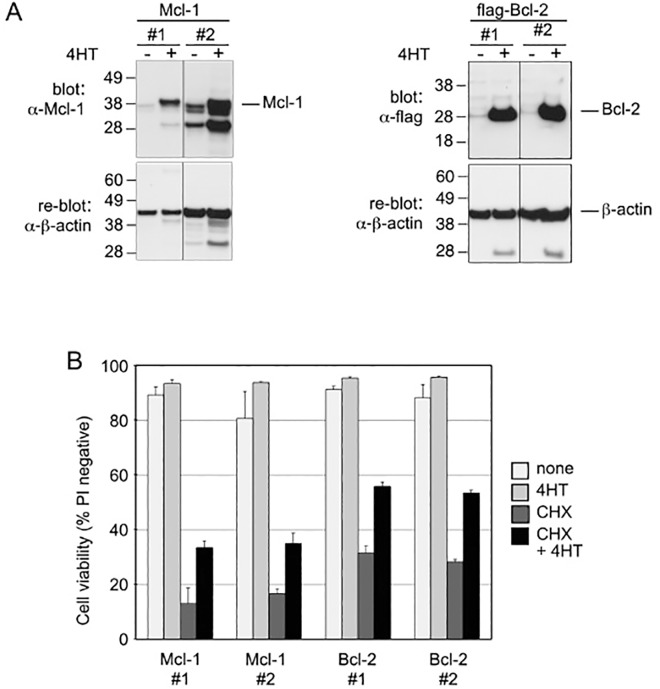
Mcl-1 over-expression gives partial protection to wild-type cells from CHX-induced cell death. A) Clones of WT FDM cells bearing inducible Mcl-1 or Bcl-2 expression constructs were treated for 24 h with or without 100 nM 4HT to induce Mcl-1 or Bcl-2. Cell lysates were prepared and immunoblotted with anti-Mcl-1, anti-flag (to detect flag-Bcl-2) or ß-actin as indicated. B) The same cells as in (A) were treated with 100 nM 4HT for 24 h before being culture in the presence or absence of 20 μg/ml CHX for a further 24 h before cell viabilities were determined by PI exclusion and flow cytometry. Data shown is expressed as mean ± SEM from 3 independent experiments. The numbers after a cell line’s name refer to independent clonal lines of the same genotype.

To confirm that lack of Mcl-1 was responsible for the death of CHX treated FDM cells, we tested whether over-expression of Mcl-1 from a 4HT-inducible construct was sufficient to prevent their death. Two independent clones of WT FDM cells were generated that each expressed either Mcl-1 or Bcl-2 following exposure to 4HT (Fig 6A). Both inducible clones expressed similar levels of Bcl-2, but the Mcl-1 clone 2 cells expressed more Mcl-1 than clone 1 cells. The over-expression of Mcl-1 had only a modest protective effect and was less effective than Bcl-2 over-expression in preventing CHX-induced cell death (Fig 6B). These results indicate that the over-expression of Mcl-1 or Bcl-2 can at least partially prevent the effect of CHX-triggered cell death.

**Fig 7:**
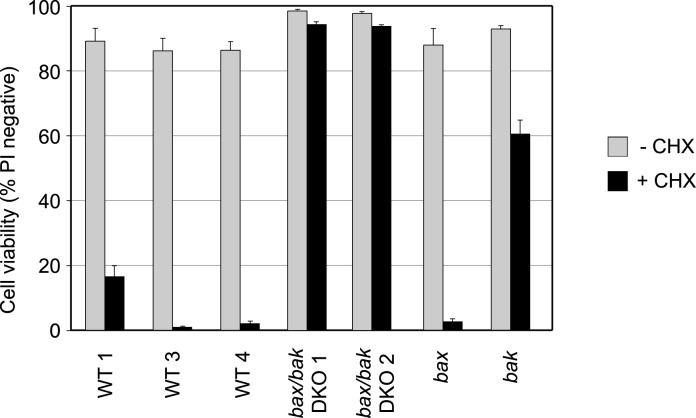
*Bak* deficient FDM cells are resistant to CHX-induced apoptosis. Cell viabilities of WT, *bax/bak* DKO, and *bax* and *bak* SKO FDM cell lines were determined after 24 h of treatment with 20 μg/ml CHX. Data shown is expressed as mean ± SEM from 3 independent experiments. The numbers after a cell line’s name refer to independent clonal lines of the same genotype.

These results indicate that cell death following addition of CHX is dependent on either Bax or Bak. To determine which of these proteins played the greater role, we generated FDM cells from mice in which the genes for either Bax or Bak were deleted. Consistent with previous results all four independent WT FDM cell lines were highly sensitive to CHX, with a significant loss in viability observed after 24 h treatment (Fig 7). Again both *bax/bak* DKO cell lines were resistant to CHX treatment. Interestingly, *bax* SKO cells were as sensitive to CHX treatment as WT cells. However, *bak* SKO cells showed marked resistance to CHX induced cell death, with 60% of cells remaining viable after 24 h CHX treatment. These results indicate that Bak, rather than Bax, plays the major role in FMD cell death following CHX treatment.

**Fig 8:**
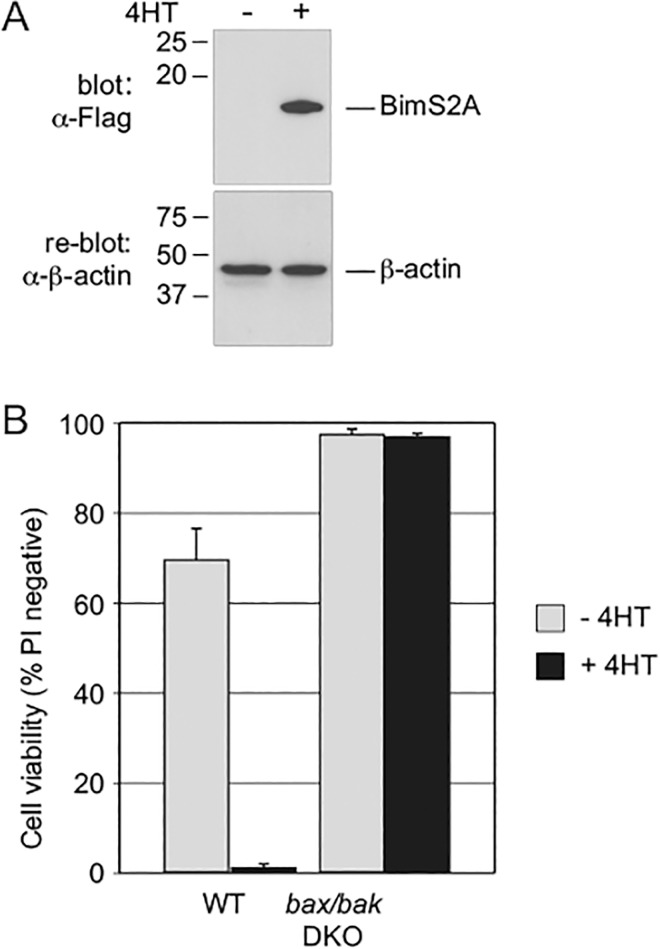
BimS2A induces efficient cell death in wild-type FDM cells. A) Inducible *bax/bak* DKO FDM cells were treated for 24 h with or without 100 nM 4HT to induce BimS2A expression as indicated. Cell lysates were prepared and immunoblotted with anti-flag to detect flag-BimS2A or ß-actin as indicated. B) Inducible WT and *bax/bak* DKO FDM cells were treated with or without 100 nM 4HT to induce BimS2A expression as indicated. Cell viabilities were determined 24 h after the addition of 4HT. Data shown is expressed as mean ± SEM from 3 independent experiments.

To further test the dependence of the FDM cells on Mcl-1, we determined the effect of antagonizing Mcl-1 by inducing expression of the engineered BH3-only protein, BimS2A, which is highly selective for Mcl-1 [14]. The addition of 4HT to *bax/bak* DKO cells bearing a 4HT-inducible construct resulted in a robust expression of the BimS2A peptide (Fig 8A) yet, as expected, it was unable to trigger death in these cells (Fig 8B). In contrast induction of BimS2A in WT cells caused all the cells to die, indicating that the functional inactivation of Mcl-1 is sufficient to induce FDM cell death.

## Discussion

It has generally been assumed that initiation of Bax/Bak dependent apoptosis requires the production or activation of one of more of the BH3-only members of the Bcl-2 family [10]. The activation of BH3-only proteins has been shown to occur via transcriptional or post-translational mechanisms in response to a number of external and intracellular signals. For example, DNA damage can promote stabilization of p53, which directly transactivates the *puma* locus [15–17], Bid can be activated by proteolytic cleavage [18], and Bim can be regulated by phosphorylation [19–22]. Once active BH3-only proteins are produced, they are believed to trigger Bax and Bak in either of two ways. The “direct activators” such as Bim, Puma and Bid can directly bind to Bax and/or Bak and cause them to form multimers in the mitochondrial outer membrane that allow exit of proteins including cytochrome c. Alternatively, the “indirect activators” such as Bad, Hrk, Bmf, and Bik bind to the anti-apoptotic Bcl-2 family members, and thereby prevent them from binding to and inhibiting Bax and Bak, and perhaps also by displacing Bim, Puma or Bid from the anti-apoptotic Bcl-2 family members so they are free to activate Bax or Bak. By either of these scenarios it is widely assumed that the BH3-only proteins perform an obligatory role in Bax/Bak activation [8, 10]. Consistent with this, mice deficient in Bim, Bid and Puma phenotypically resemble *bax/bak* DKO mice, suggesting they are essential for Bax/Bak activation [9].

Here we provide evidence that apoptosis can be triggered in another way, in the absence of BH3-only proteins Blk, Bad, Hrk, or Noxa, and in cells where none of the known direct activator BH3-only proteins (Bid, Bim, Puma) are present at detectable levels. Our results in IL-3 dependent myeloid lines suggest that without triggering by BH3-only proteins, Bax and Bak can spontaneously activate when levels of anti-apoptotic Bcl-2 family proteins decline. Consistent with our findings, Muer *et al*. reported that expression of p14^ARF^ could induce Bak dependent apoptosis of DU145 prostate carcinoma cells by down regulation of Mcl-1 and Bcl-xl without the activation of BH3-only proteins [23]. Similarly, Chen and colleagues showed in MEFs that Bax and Bak auto-activated in the absence of BH3-only proteins when Bcl-2, Bcl-xl and Mcl-1 levels decreased following treatment with etoposide [24]. Moreover, in a *tour de force* involving simultaneous mutation of the genes for eight BH3-only proteins, five anti-apoptotic Bcl-2 family proteins, as well as Bax and Bak in HCT116 colorectal carcinoma cells, O'Neill *et al*. showed that Bax or Bak are able to associate with the outer mitochondrial membrane, activate, and cause apoptosis spontaneously, without being activated by any BH3-only protein [25].

CHX-induced death of mouse FDM cells required Bax and/or Bak, because cells lacking both Bax and Bak were profoundly resistant to CHX (although even *bax/bak* DKO FDM cells do eventually die if CHX is never removed from their media). Cells deficient in *bak* only were highly resistant to CHX-induced apoptosis, but not as resistant as *bax/bak* deficient cells. In contrast, *bax* deficient FDM cells were as sensitive to the effects of CHX as WT cells. These results indicate that CHX-triggered apoptosis of these cells is predominantly mediated by Bak, and in its absence Bax can only induce limited amounts of cell death.

These results are consistent with studies using baby mouse kidney (BMK) cells, and cells that lacked Bax and Bak [26], which were also highly resistant to CHX. Interestingly, *bax* deficient BMK cells that retained one allele of *bak* were as sensitive to CHX as WT cells, yet *bak* deficient cells that retained one copy of *bax* were as resistant to CHX as cells that lacked both *bax* and *bak* [26] indicating that Bak is the main mediator of death in these cells, as it was in the FDMs.

The common requirement for Bak in CHX-induced death in both FDM and BMK cells suggested that a similar mode of activation might occur in the two cell types. However, BMK cells that lacked Blk (Bik in humans) were also highly resistant to CHX indicating Blk is required for Bak activation in these cells [26]. A similar essential requirement for Bim in CHX-induced death has also been demonstrated in HeLa cells [27]. In contrast to these studies none of the BH3-only proteins were found to be necessary for CHX-induced FDM cell death. None of the SKO lines that lacked a single member of the BH3-only family exhibited any resistance towards CHX. Similarly, none of the DKO lines that were deficient in *bad/bim*, *bid/bim* or *puma/noxa* displayed any resistance towards CHX. Complementing these findings, roscovitine also lead to cell death in a *bax/bak* dependent manner, resulted in the loss of Mcl-1, and the BH3-only SKO and DKO cell lines did not show any protection against cell death. Using this eukaryotic model, it was not possible to knockout all the BH3-only proteins simultaneously, however the SKO and DKO lines tested do indicate that CHX is not inducing cell death through any single known BH3-only protein. Furthermore, the presence of CHX would prevent the synthesis of new BH3-only proteins (such as Puma or Noxa) to activate Bak to cause cell death. This finding supports a model in which several known BH3-only proteins are not involved in CHX-induced FDM cell death. This finding is further supported by our results using roscovitine which, like CHX, caused loss of Mcl-1 and induced Bax/Bak dependent cell death. Moreover, the finding that none of the BH3-only knockout cell lines provided protection to roscovitine treatment suggests that known BH3-only proteins are also not involved in roscovitine-induced FDM cell death.

Our results suggest that as Mcl-1 levels decline, Bax/Bak can activate spontaneously, without being activated by a ‘direct’ activator BH3-only protein, such as Bim, Puma or Bid. While it has been reported that over-expression of Bax or Bak in yeasts and mammalian cells can lead to their spontaneous activation [28–30], in our experiments Bax and Bak existed at their physiological levels.

Mcl-1 is unique among members of the Bcl-2 pro-survival family in that it is rapidly turned over by the action of ubiquitin ligases (such as Mule) [31–33] and can be stabilised by the action of deubiquitinases (e.g. USP9X) [34]. Consistent with this, Mcl-1 abundance was rapidly decreased to barely detectable levels in FDM cells within 4 h of CHX addition, whereas none of the other Bcl-2 family members showed any appreciable decrease over the time-course examined. Furthermore, the treatment with the proteasome inhibitor, MG132, increased abundance of Mcl-1 in resting cells and prevented the loss of Mcl-1 following CHX addition. Thus the normal proteasomal turnover of Mcl-1 can account for the loss of Mcl-1 following inhibition of protein synthesis with CHX.

Bak is predominantly inhibited by the actions of Mcl-1 and Bcl-xL and not other pro-survival family members [26, 35]. Thus Mcl-1 loss in FDM cells following protein synthesis inhibition is consistent with the observed requirement of Bak to mediate CHX-induced cell death. However, it is worth noting the *bak* deficient FDM cells were not completely resistant to CHX like *bax/bak* deficient cells. Over-expression of Mcl-1 and Bcl-2 both increased cell survival after CHX-treatment. Since Mcl-1 is rapidly turned over, its over-expression did not raise its levels as much as over-expression of Bcl-2, which is more stable, and hence over-expression of Bcl-2 gave a greater level of protection (Fig 6).

Loss of Mcl-1 has been observed previously in HeLa cells [27] and in other cell types including leukemic cells and fibroblasts [36] following inhibition of protein synthesis. However, in contrast to the results here, it was concluded in these studies that Mcl-1 loss was not the sole event to trigger cell death [27, 36]. Indeed, in HeLa cells Bim was also required for CHX-induced cell death [27] and Bim can inhibit all pro-survival family proteins [37]. Furthermore, in CHX treatment of BMK cells it was shown that Blk was required to antagonise the functions of Mcl-1 and Bcl-xL to cause death [26].

The results presented here are consistent with a model in which a decline in Mcl-1 levels is the key event leading to Bak activation and cell death following protein synthesis inhibition. This model predicts that the specific inactivation of Mcl-1 should be sufficient to cause cell death. In support of this the over-expression of BimS2A, which specifically binds and inhibits the function of Mcl-1, efficiently induced cell death in WT but not *bax/bak* deficient FDM cells.

In conclusion none of the tested BH3-only proteins were found to be required for CHX-induced or roscovitine-induced FDM cell death. Rather the loss of Mcl-1 was the key event leading to Bak activation and cell death. The results of this study are consistent with a model in which there is no obligatory requirement for the direct activation of Bak by several known BH3-only proteins in CHX-induced apoptosis in FDM cells.

## Materials and Methods

### Antibodies and chemicals

Mouse monoclonal anti-flag (M2 clone; #F1804) and anti-ß-actin (clone AC-15; #A1978) were purchased from Sigma (Castle Hill, NSW). Rabbit polyclonal anti-Mcl-1 (#600-401-394) was purchased from Rockland (Jomar Bioscience, Welland, SA). Hamster monoclonal anti-Bcl-2 (clone 3F11) was obtained from Andreas Strasser (WEHI). Anti-Bcl-w and anti-Bcl-xL were obtained from Lorraine O'Reilly (WEHI) and rat anti-A1 (clone 6D6) was provided by David Huang (WEHI). Cycloheximide (#C7698), 4-hydroxytamoxifen (4HT; #H7904), roscovitine (#R7772) and MG132 (#C2211) were purchased from Sigma.

### Plasmids and cDNAs

MmMcl-1 and HsBcl-2 cDNAs were generously provided by David Huang (WEHI) while BimS2A cDNA was provided by Doug Fairlie (WEHI). BamHI/XbaI fragments or compatible fragments containing their respective coding regions were subcloned in frame into the 4HT-inducible vector, pF-5xUAS-flag-W-SV40puro [38], to generate N-terminally flag-tagged constructs.

### Cell Culture

#### Generation of IL-3 dependent cell lines

Factor dependent myeloid (FDM) cells were generated from E14.5 mice, by co-culturing single cell suspensions from fetal liver with fibroblasts producing a HoxB8 expressing retrovirus in the presence of 0.25 ng/ml recombinant mouse IL-3 (R&D Systems, Sapphire Biosciences, Redfern, NSW). *Noxa*^*-/-*^, *Bmf*^*-/-*^, *Hrk*^*-/-*^, *Bik*^*-/-*^ and *Puma*^*-/-*^*;Noxa*^*-/-*^ mice have been described before [39–43]. All mice were derived from C57BL/6 embryonic stem (ES) cells or from 129SV-derived ES cells and back- crossed onto a C57BL/6 background for at least 10 generations. Cell lines were tested for IL-3 dependence by determining their ability to proliferate in the absence of IL-3 in soft agar. No cell lines were able to generate colonies under such conditions. Wild-type FDM cells and *Bad*^*-/-*^, *Bid*^*-/-*^, *Bim*^*-/-*^, *Puma*^*-/-*^, *Bax*^*-/-*^, *Bak*^*-/-*^, *Bax*^*-/-*^*;Bak*^*-/-*^, *Bad*^*-/-*^*;Bim*^*-/-*^ and *Bid*^*-/-*^*;Bim*^*-/-*^ FDM cells have all been described previously [2, 4]. All cells were cultured in DMEM (low glucose; Life Technologies, Melbourne, VIC) supplemented with 10% fetal bovine serum (Life Technologies), 0.25 ng/ml IL-3, penicillin G 50 U/ml/streptomycin (50 μg/ml), L-glutamine (2 mM) in a humidified atmosphere of 10% CO_2_ at 37°C. For IL-3 withdrawal experiments cells were washed at least 3 times with phosphate buffered saline (PBS) before being resuspended in complete media without IL-3.

#### Generation of 4-hydroxytamoxifen-inducible cell lines

Lentiviruses were first prepared by transfecting 293T cells with the appropriate lentiviral vector together with pCMV-ΔR8 and pVSV-G packaging constructs using Effectene as described previously [38]. 48 h after transfection viral supernatants were filtered, mixed with polybrene (4 μg/mL), and added to target cells which were then centrifuged at 1 250 g for 90 min at room temperature. Stably infected 4HT-inducible cell lines were selected in the presence of puromycin and hygromycin B. Gene expression in target cells was induced by the addition of 100 nM 4HT.

### Cell viability assays

Cells were centrifuged at 1 700 rpm (415 g) for 5 min at 4°C and re-suspended in 100–200 μl PBS containing 1 μg/ml of propidium iodide (PI) and sorted using a FACSCalibur flow cytometer (Becton Dickinson, North Ryde, New South Wales). Twenty thousand events per sample were collected and analyzed using WEASEL software (version 2.2.2, WEHI). Viable cells were identified by their ability to exclude propidium iodide. Unless otherwise stated, all cell death/viability experiments were independently repeated at least three times.

### Cell lysis and immunoblotting

Cells were centrifuged at 1 700 rpm for 5 min and washed with ice-cold PBS. Cells were lysed directly in sample buffer (100 mM Tris-Cl pH 6.8, 4% (w/v) SDS, 20% (v/v) glycerol) and boiled for 10 min. Alternatively, cells were re-suspended in DISC lysis buffer [44] supplemented with complete protease inhibitor cocktail (Roche), 10 mM NaF, 2 mM Na pyrophosphate, 1 mM Na molybdate and 5 mM ß-glycerophosphate, incubated on ice for 30–60 min and clarified by centrifugation at 13 000 g for 10 min at 4°C. All lysates were stored at -80°C. Clarified lysates (50 μg) were mixed with sample buffer, boiled and separated by sodium dodecyl sulphate polyacrylamide gel electrophoresis (SDS-PAGE) on linear gels or 4–20% Tris-glycine gradient gels (Bio-Rad, Gladesville, NSW) or 4–12% Bis-Tris gradient NuPage gels (Life Technologies) and transferred to Hybond-C nitrocellulose membrane (GE, Castle Hill, NSW). Membranes were blocked in 5% skim milk powder in Tris-buffered saline containing 0.1% Tween-20 (TBST) or PBS containing 0.1% Tween-20 (PBST) and incubated with primary antibody overnight at 4°C or for 2 hours at room temperature. Membranes were washed four times with TBST or PBST at room temperature, incubated with horseradish peroxidase conjugated secondary antibody (GE) and washed before detection with enhanced chemiluminescence. Where necessary, membranes were stripped in buffer (100 mM Tris adjusted to pH 7.4, 2% SDS, 350 μl/50 ml β-mercaptoethanol) incubated for 15 min at 60°C. The membrane was rinsed thoroughly with dH_2_O (3 x 20 ml) then rinsed three times with TBST before being re-blocked with milk.

## Abbreviations

PI: propidium iodide
FDM: Factor dependent myeloid
ES: embryonic stem
PBS: phosphate buffered saline
TBST: Tris-buffered saline containing 0.1% Tween-20
4HT: 4-hydroxytamoxifen
CHX: cycloheximide
DKO: double knockou
SKO: single knockout
